# Associação entre a Ocorrência de Fibrilação Atrial e a Síndrome de Wolff-Parkinson-White: A Predição é Possível?

**DOI:** 10.36660/abc.20260001

**Published:** 2026-06-10

**Authors:** Afonso Dalmazio Souza Mario, Eduardo Pelegrineti Targueta, Francisco Carlos da Costa Darrieux, Alexandra Regia Dantas Brigido, Alberto Pereira Ferraz, Muhieddine Omar Chokr, Cristiano F. Pisani, Mauricio Ibrahim Scanavacca

**Affiliations:** 1 Instituto do Coração do Hospital das Clínicas da Faculdade de Medicina da Universidade de São Paulo São Paulo SP Brasil Instituto do Coração do Hospital das Clínicas da Faculdade de Medicina da Universidade de São Paulo, São Paulo, SP – Brasil

**Keywords:** Fibrilação Atrial, Síndrome de Wolff-Parkinson-White, Eletrofisiologia, Arritmias Cardíacas

## Abstract

**Fundamento:**

Pacientes com síndrome de Wolff-Parkinson-White (WPW) frequentemente desenvolvem fibrilação atrial (FA); no entanto, os fatores associados à ocorrência de FA permanecem incompletamente definidos.

**Objetivos:**

Avaliar a prevalência e os fatores associados à FA clínica e à FA induzida durante o estudo eletrofisiológico (EEF) em uma grande coorte consecutiva de pacientes com WPW submetidos à ablação de via acessória (VA).

**Métodos:**

Esta análise retrospectiva incluiu 845 pacientes consecutivos com WPW submetidos à ablação de VA em um único centro terciário de referência. Um protocolo padronizado de EEF foi aplicado durante todo o período do estudo. Os desfechos incluíram FA clínica (documentada por histórico médico ou monitorização antes do EEF), FA induzida no EEF e qualquer FA (desfecho composto). Modelos de regressão logística multivariável foram utilizados para estimar *odds ratios* ajustados (OR_a_) com intervalo de confiança de 95% (IC 95%). A significância estatística foi definida como p < 0,05.

**Resultados:**

A média de idade foi de 33,2 ± 15,9 anos, e 486 dos 845 pacientes (57,5%) eram do sexo masculino. Múltiplas VAs foram identificadas em 31 pacientes (3,7%). As localizações mais comuns das VAs foram lateral esquerda (327/845, 38,7%) e posterior (323/845, 38,2%). FA clínica esteve presente em 109 pacientes (12,9%), FA induzida no EEF em 77 (9,1%) e qualquer FA em 168 (19,9%). Nas análises multivariáveis, uma VA lateral esquerda esteve independentemente associada à FA clínica (OR_a_, 2,31; intervalo de confiança de 95% [IC 95%], 1,53-3,49; p < 0,001), enquanto uma VA posterior esteve associada à FA induzida no EEF (OR_a_, 1,78; IC 95%, 1,07-2,91; p = 0,025). O sexo feminino esteve associado a menores chances de FA induzida no EEF (OR_a_, 0,56; IC 95%, 0,34-0,93; p = 0,025). O aumento da idade esteve independentemente associado à FA clínica (OR_a_ por ano, 1,018; p = 0,007) e a qualquer FA (OR_a_ por ano, 1,014; p = 0,009).

**Conclusão:**

Nesta grande coorte de pacientes com WPW submetidos à ablação de VA, a localização lateral esquerda da VA e a maior idade estiveram independentemente associadas a maior prevalência de FA clínica. Em contraste, a localização posterior da VA e o sexo masculino estiveram associados a maior probabilidade de FA induzida no EEF.

## Graphical abstract

**Figure:**
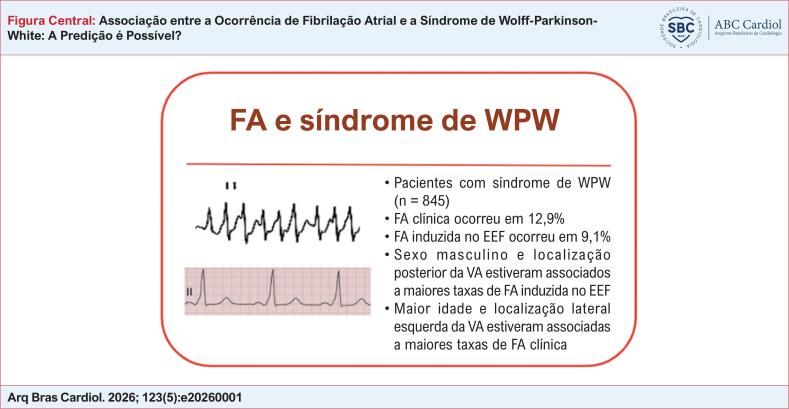
EEF: estudo eletrofisiológico; FA: fibrilação atrial; VA: via acessória; WPW: Wolff-Parkinson-White.

## Introdução

A fibrilação atrial (FA) é uma das manifestações arrítmicas observadas em pacientes com síndrome de Wolff-Parkinson-White (WPW).^1^ Entretanto, a FA nessa população apresenta relevância clínica devido à sua potencial associação com morte cardíaca súbita.^2^ Diversos mecanismos têm sido propostos para explicar a ocorrência de FA em pacientes com WPW, incluindo a degeneração da taquicardia por reentrada atrioventricular (TRAV) em FA, as propriedades eletrofisiológicas da via acessória (VA), os efeitos da VA sobre a arquitetura atrial e a vulnerabilidade intrínseca do miocárdio atrial.

Os impulsos elétricos que alcançam os átrios por meio de uma VA durante a TRAV podem desencadear episódios de FA. Além disso, a TRAV pode aumentar a vulnerabilidade atrial por meio da intensificação da ativação simpática e do estiramento das fibras atriais causado pelas alterações hemodinâmicas associadas à taquiarritmia.^3^ A redução da incidência de FA após a ablação de VA por cateter reforça ainda mais o papel da VA na fisiopatologia dessa arritmia.^4,5^ Algumas características clínicas, incluindo idade mais avançada, sexo masculino e localização posterosseptal da VA, têm sido associadas a uma maior probabilidade de ocorrência de FA.^6^

Expandir as evidências disponíveis sobre a ocorrência de FA em pacientes com WPW é importante e pode contribuir para futuras metanálises. Portanto, este estudo teve como objetivo avaliar os fatores associados à ocorrência de FA em pacientes com WPW por meio de um estudo de associação baseado em uma coorte histórica.

## Métodos

### Delineamento do estudo e população

Este estudo retrospectivo, analítico e de associação (coorte histórica) foi conduzido em conformidade com os princípios da Declaração de Helsinque e aprovado pelo comitê de ética institucional local.

**Figura 1:**
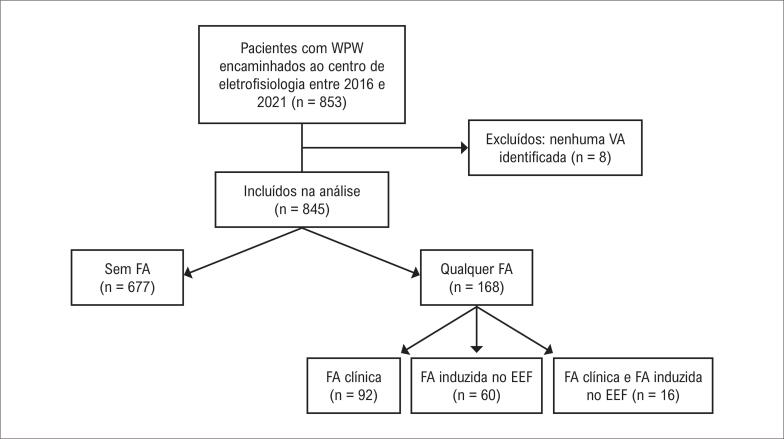
Fluxograma da população do estudo com síndrome de WPW. EEF: estudo eletrofisiológico; FA: fibrilação atrial; VA: via acessória; WPW: Wolff-Parkinson-White.

Foram incluídos todos os pacientes consecutivos com síndrome de WPW submetidos à ablação de VA entre janeiro de 2016 e agosto de 2021 em um centro terciário de referência em cardiologia (Figura 1). Os critérios de inclusão exigiram a conclusão do procedimento de ablação. As indicações para ablação incluíram TRAV sintomática ou pacientes assintomáticos com propriedades de condução de alto risco. Esta coorte foi independente de qualquer conjunto de dados previamente publicado.

### Protocolo do estudo eletrofisiológico

A estimulação atrial programada foi realizada a partir do seio coronariano (SC) proximal utilizando ciclos básicos de 600, 500 e 400 ms, com até dois extraestímulos atriais administrados até o período refratário efetivo ou um intervalo mínimo de acoplamento de 200 ms, seguidos de estimulação decremental. Um protocolo padronizado de indução foi aplicado de forma consistente durante todo o período do estudo.

O protocolo foi repetido após administração intravenosa de isoproterenol (bolus de 10-20 mcg). A FA induzida incluiu tanto FA desencadeada intencionalmente durante a estimulação programada quanto início inadvertido de FA durante o procedimento. A FA foi definida como ≥ 10 segundos de atividade atrial irregular sem morfologia organizada de taquicardia atrial (TA) ou TRAV. A FA ocorrida exclusivamente durante a manipulação do cateter foi classificada como FA induzida apenas quando reproduzível por estimulação atrial programada.

### Desfechos

Os desfechos do estudo incluíram FA clínica, FA induzida durante o estudo eletrofisiológico (EEF) e qualquer FA (desfecho composto incluindo FA clínica ou induzida).

### Análise estatística

As variáveis contínuas são apresentadas como média ± desvio padrão, enquanto as variáveis categóricas são expressas como frequências absolutas (n) e relativas (%).

A frequência de cada apresentação de FA (FA clínica, FA induzida no EEF ou qualquer FA) foi avaliada de acordo com variáveis qualitativas. As associações entre variáveis categóricas foram avaliadas pelo teste do qui-quadrado ou teste exato de Fisher, conforme apropriado. As distribuições de idade de acordo com a ocorrência de FA foram comparadas utilizando o teste *t* de Student não pareado.

Os *odds ratios* (OR) para cada desfecho relacionado à FA foram inicialmente estimados por modelos de regressão logística univariável. Posteriormente, modelos de regressão logística multivariável foram construídos para identificar preditores independentes de cada desfecho relacionado à FA. Sexo, idade e características procedimentais com p < 0,20 nas análises não ajustadas foram incluídos nos modelos multivariáveis. Variáveis com p < 0,20 na análise univariável foram selecionadas de acordo com recomendações estabelecidas para modelagem por regressão logística, a fim de evitar a exclusão prematura de potenciais preditores relevantes.^7^

A normalidade dos dados foi avaliada pelo teste de Shapiro-Wilk, e todas as variáveis contínuas apresentaram distribuição normal. As análises estatísticas foram realizadas utilizando o IBM SPSS Statistics for Windows, versão 22.0 (IBM Corp., Armonk, NY, EUA). O Microsoft Excel 2013 (Microsoft Corp., Redmond, Washington, EUA) foi utilizado para tabulação dos dados. Um valor de p bicaudal < 0,05 foi considerado estatisticamente significativo.

## Resultados

A coorte do estudo compreendeu 845 pacientes com síndrome de WPW submetidos à ablação de VA por cateter. As características basais estão resumidas na Tabela 1. A média de idade foi de 33,2 ± 15,9 anos, e 139 pacientes (16,4%) tinham menos de 18 anos. No total, 486 pacientes eram do sexo masculino (57,5%).

**Tabela 1:** Características basais dos pacientes e achados procedimentais

Variável		Valor
Pacientes		845
Sexo feminino		359 (42,5)
Faixa etária, anos		33,2 ± 15,9
	< 18 anos	139 (16,4)
	18-39 anos	431 (51,0)
	40-59 anos	216 (25,6)
	≥ 60 anos	59 (7,0)
Múltiplas VAs		31 (3,7)
VA posterior		323 (38,2)
VA lateral esquerda		327 (38,7)
FA induzida no EEF		77 (9,1)
FA clínica		109 (12,9)
Qualquer FA		168 (19,9)

Os valores são apresentados como n (%) ou média ± desvio padrão. EEF: estudo eletrofisiológico; FA: fibrilação atrial; VA: via acessória.

**Figura 2:**
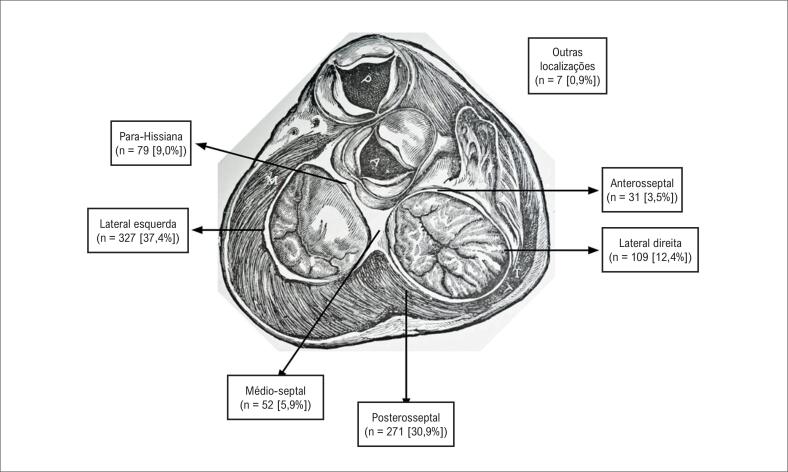
Distribuição das localizações das VAs identificadas durante o EEF em 845 procedimentos de ablação. Alguns pacientes apresentaram múltiplas VAs, resultando em um total de 876 VAs identificadas. A: valva aórtica; M: valva mitral; P: valva pulmonar; T: valva tricúspide; VA: via acessória.

As localizações mais comuns das VAs foram lateral esquerda (37,4%), póstero-septal (30,9%) e lateral direita (12,4%) (Figura 2). Múltiplas VAs foram identificadas em 31 pacientes (3,7%).

A prevalência de FA clínica foi de 12,9% (n = 109). A prevalência de FA induzida durante o EEF foi de 9,1%.

A frequência de FA aumentou progressivamente entre as faixas etárias, independentemente da definição de FA utilizada. A prevalência de FA induzida no EEF aumentou de 5,0% entre pacientes com idade < 18 anos para 15,3% entre aqueles com idade ≥ 60 anos. Da mesma forma, a prevalência de FA clínica aumentou de 9,4% para 18,6%, enquanto a prevalência de qualquer FA aumentou de 13,7% para 23,7% entre os mesmos estratos etários (Tabela 2).

**Tabela 2:** Fibrilação atrial induzida no estudo eletrofisiológico, fibrilação atrial clínica e qualquer fibrilação atrial de acordo com a faixa etária

Faixa etária, anos	FA induzida no EEF, n (%)		FA clínica, n (%)		Qualquer FA, n (%)		Total
	Não	Sim	Não	Sim	Não	Sim	
< 18	132 (95,0)	7 (5,0)	126 (90,6)	13 (9,4)	120 (86,3)	19 (13,7)	139
18-39	390 (90,5)	41 (9,5)	382 (88,6)	49 (11,4)	346 (80,3)	85 (19,7)	431
40-59	196 (90,7)	20 (9,3)	180 (83,3)	36 (16,7)	166 (76,9)	50 (23,1)	216
≥ 60	50 (84,7)	9 (15,3)	48 (81,4)	11 (18,6)	45 (76,3)	14 (23,7)	59
Total	768 (90,9)	77 (9,1)	736 (87,1)	109 (12,9)	677 (80,1)	168 (19,9)	845

A análise estratificada por idade demonstrou aumento progressivo da frequência de FA com o avanço da idade em todas as definições de FA. EEF: estudo eletrofisiológico; FA: fibrilação atrial.

Sexo e localização posterior da VA estiveram significativamente associados à FA induzida no EEF nas análises não ajustadas (p < 0,05). Após ajuste multivariável, ambas as variáveis permaneceram independentemente associadas à FA induzida (Tabela 3). O sexo feminino esteve associado a uma probabilidade 44% menor de FA induzida em comparação ao sexo masculino, enquanto a localização posterior da VA esteve associada a uma probabilidade 78% maior de FA induzida, independentemente das demais características avaliadas.

**Tabela 3:** Fibrilação atrial induzida no estudo eletrofisiológico de acordo com as características dos pacientes e procedimentais e resultados das análises univariável e multivariável

Variável	FA induzida no EEF, n (%)		OR_na_	IC 95%	Valor de p	OR_a_	IC 95%	Valor de p
	Não	Sim						
Sexo feminino, n (%)	335 (93,3)	24 (6,7)	0,590	0,350-0,970	0,035	0,560	0,340-0,930	0,025
Idade, anos (média ± DP)	32,9 ± 16,0	36,5 ± 14,8	1,014	0,999-1,029	0,060†	1,014	0,999-1,029	0,064
Múltiplas VAs, n (%)	27 (87,1)	4 (12,9)	1,500	0,510-4,420	0,517*	—	—	—
VA posterior, n (%)	283 (87,6)	40 (12,4)	1,850	1,160-2,970	0,009	1,780	1,110-2,860	0,017
VA lateral esquerda, n (%)	299 (91,4)	28 (8,6)	0,900	0,550-1,460	0,659	—	—	—

As análises ajustadas foram realizadas por meio de regressão logística multivariável.

* Teste exato de Fisher.

† Teste t de Student. DP: desvio padrão; EEF: estudo eletrofisiológico; FA: fibrilação atrial; IC 95%: intervalo de confiança de 95%; OR_a_: odds ratio ajustado; OR_na_: odds ratio não ajustado; VA: via acessória.

Conforme apresentado na Tabela 4, tanto a idade quanto a localização lateral esquerda da VA estiveram significativamente associadas à FA clínica nas análises não ajustadas e ajustadas (p < 0,05). Cada ano adicional de idade esteve associado a um aumento de 1,8% na probabilidade de FA clínica. Além disso, pacientes com VA lateral esquerda apresentaram probabilidade 2,31 vezes maior de FA clínica em comparação àqueles sem essa localização de VA, independentemente das demais características avaliadas.

**Tabela 4:** Fibrilação atrial clínica de acordo com as características dos pacientes e procedimentais e resultados das análises univariável e multivariável

Variável	FA clínica, n (%)		OR_na_	IC 95%	Valor de p	OR_a_	IC 95%	Valor de p
	Não	Sim						
Sexo feminino, n (%)	313 (87,2)	46 (12,8)	0,990	0,660-1,480	0,949	0,930	0,620-1,420	0,748
Idade, anos (média ± DP)	32,6 ± 15,6	37,7 ± 16,8	1,020	1,008-1,033	0,001^†^	1,018	1,005-1,031	0,007
Múltiplas VAs, n (%)	28 (90,3)	3 (9,7)	0,720	0,210-2,400	0,787*	—	—	—
VA posterior, n (%)	284 (87,9)	39 (12,1)	0,890	0,580-1,350	0,574	—	—	—
VA lateral esquerda, n (%)	264 (80,7)	63 (19,3)	2,450	1,630-3,690	< 0,001	2,310	1,530-3,490	< 0,001

As análises ajustadas foram realizadas por meio de regressão logística multivariável.

* Teste exato de Fisher.

† Teste t de Student. DP: desvio padrão; FA: fibrilação atrial; IC 95%: intervalo de confiança de 95%; OR_a_: odds ratio ajustado; OR_na_: odds ratio não ajustado; VA: via acessória.

Achados semelhantes foram observados para qualquer FA (Tabela 5). Após ajuste, cada ano adicional de idade aumentou a probabilidade de qualquer FA em 1,4%, enquanto pacientes com VA lateral esquerda apresentaram probabilidade 1,65 vez maior de qualquer FA em comparação àqueles sem essa localização de VA, independentemente das covariáveis remanescentes.

**Tabela 5:** Qualquer fibrilação atrial de acordo com as características dos pacientes e procedimentais e resultados das análises univariável e multivariável

Variável	Qualquer FA, n (%)		OR_na_	IC 95%	Valor de p	OR_a_	IC 95%	Valor de p
	Não	Sim						
Sexo feminino, n (%)	296 (82,5)	63 (17,5)	0,770	0,550-1,090	0,144	0,740	0,520-1,050	0,088
Idade, anos (média ± DP)	32,5 ± 15,8	36,3 ± 15,8	1,015	1,004-1,026	0,005†	1,014	1,004-1,025	0,009
Múltiplas VAs, n (%)	26 (83,9)	5 (16,1)	0,770	0,290-2,030	0,594	—	—	—
VA posterior, n (%)	253 (78,3)	70 (21,7)	1,200	0,850-1,690	0,305	—	—	—
VA lateral esquerda, n (%)	244 (74,6)	83 (25,4)	1,730	1,230-2,440	0,001	1,650	1,170-2,330	0,004

As análises ajustadas foram realizadas por meio de regressão logística multivariável.

* Teste exato de Fisher.

† Teste t de Student. DP: desvio padrão; FA: fibrilação atrial; IC 95%: intervalo de confiança de 95%; OR_a_: odds ratio ajustado; OR_na_: odds ratio não ajustado; VA: via acessória.

Os principais achados são resumidos na Figura Central. As análises de sensibilidade excluindo pacientes com múltiplas VAs mostraram resultados amplamente consistentes. A única alteração relevante foi que o sexo feminino se tornou limítrofe para significância estatística em relação à FA induzida no EEF após a exclusão de pacientes com múltiplas VAs (OR ajustado, 0,617; intervalo de confiança de 95%, 0,370-1,028; p = 0,064).

## Discussão

Até onde sabemos, esta representa a terceira maior coorte contemporânea de pacientes com síndrome de WPW submetidos à ablação de VA por cateter relatada até o momento.^8–10^

A média de idade da nossa coorte foi de 33 anos, superior aos 19 anos relatados por Pappone et al.^9^ em 2014. Essa diferença pode refletir disparidades no acesso a centros especializados de referência em arritmias no Brasil.

Múltiplas VAs foram identificadas em 3,7% dos pacientes, uma proporção inferior à previamente relatada em outras coortes contemporâneas.^8–10^ Ainda assim, a distribuição das VAs foi consistente com estudos anteriores,^9^ sendo as VAs laterais esquerdas as mais comuns, seguidas pelas VAs posteroseptais.

Nossos achados em relação à prevalência de FA induzida no EEF também foram comparáveis aos relatados em pesquisas anteriores (9,1% vs 11,1%).^9^ Da mesma forma, a prevalência de FA clínica em nossa coorte (12,9%) excedeu a prevalência de 2%-4% relatada para a população geral, mas foi semelhante à observada em outros registros de ablação de WPW (10% e 12,8%).^8,10,11^

Em nossa coorte, a localização posterior da VA esteve associada a maior probabilidade de FA induzida no EEF, enquanto a localização lateral esquerda da VA esteve associada à FA clínica. Diversos mecanismos podem explicar a associação entre a localização da VA e a ocorrência de FA. Diferenças anatômicas na inserção da VA podem contribuir para instabilidade elétrica atrial. VAs posteriores, que frequentemente apresentam padrão de inserção oblíquo, podem alterar a arquitetura miocárdica atrial e aumentar a suscetibilidade à arritmogênese atrial.^3^

Em relação às VAs esquerdas, estudos prévios descreveram associação entre VAs laterais esquerdas e potenciais duplos (PDs) atriais. Os PDs são tipicamente identificados durante registros de TRAV no SC. Um estudo de coorte polonês publicado em 2017 demonstrou associação entre PDs atriais, FA clínica, FA induzida e idade mais avançada.^12^ No estudo, pacientes com PDs atriais também apresentaram frequências cardíacas mais elevadas durante a TRAV e maior alternância elétrica.

Um possível mecanismo subjacente a essa associação é a ativação simultânea em direção tanto ao SC distal, próximo à veia pulmonar superior esquerda ou ao anel mitral, quanto ao SC proximal (óstio), levando à ativação do nó atrioventricular. A geração de frentes de onda atriais duais pode ser um fator para o início da FA. Além disso, o SC possui estreitas relações anatômicas com o átrio direito inferoposterior e o átrio esquerdo posterior, estruturas reconhecidamente envolvidas na manutenção da FA. O comprimento do ciclo da taquicardia também pode contribuir para a vulnerabilidade atrial, uma vez que ciclos mais curtos podem facilitar o início da FA ao reduzir os períodos refratários atriais.

De forma semelhante à TRAV, extrassístoles ventriculares com condução retrógrada também têm sido associadas à ocorrência de FA. Evidências prévias sugerem que múltiplas VAs aumentam o risco de FA, provavelmente por intensificarem a ativação atrial retrógrada. Entretanto, essa associação não foi observada em nossa coorte, possivelmente devido à prevalência relativamente baixa de múltiplas VAs em nossa população.

As propriedades de condução anterógrada também têm sido relacionadas à ocorrência de FA.^1^ Estudos anteriores demonstraram maior prevalência de FA em pacientes com VAs manifestas em comparação àqueles com VAs ocultas. Além disso, características eletrofisiológicas da VA, particularmente períodos refratários mais curtos, têm sido associadas a maior probabilidade de FA.

Interpretamos a FA induzida no EEF como um teste fisiológico de estresse refletindo vulnerabilidade atrial, e não como um preditor determinístico de FA espontânea em nível individual. A divergência observada em nosso estudo, com a FA clínica associada predominantemente às VAs laterais esquerdas e a FA induzida no EEF associada às VAs posteriores, pode refletir mecanismos distintos de gatilho e substrato, incluindo dispersão atrial esquerda por frentes de onda duais versus gatilhos eletricamente lábeis e facilitados por estímulo adrenérgico próximos ao SC e às regiões inferosseptais.

### Limitações do estudo

Este estudo apresenta várias limitações. Primeiramente, por se tratar de um estudo retrospectivo unicêntrico, viés de seleção não pode ser excluído, apesar do grande tamanho amostral. Informações detalhadas sobre as categorias de indicação da ablação (pacientes sintomáticos versus pacientes assintomáticos de alto risco) não estavam consistentemente disponíveis, embora todos os pacientes incluídos tenham sido submetidos à ablação por uma dessas indicações. O *status* de VA manifesta versus oculta também não foi sistematicamente registrado e, portanto, não pôde ser incorporado às análises. Além disso, o comprimento do ciclo da TRAV não foi consistentemente documentado durante todo o período do estudo, impossibilitando análises envolvendo a frequência da taquicardia.

A associação entre localização lateral esquerda da VA e FA clínica também deve ser interpretada com cautela, particularmente porque as VAs laterais esquerdas representaram a localização de VA mais prevalente em nossa coorte e porque a idade é um determinante forte e universal do risco de FA. Por fim, o seguimento em longo prazo após a ablação não estava disponível, o que limita nossa capacidade de determinar se a vulnerabilidade atrial na WPW representa um fenômeno eletrofisiológico transitório ou um substrato independente em longo prazo para o desenvolvimento futuro de FA.

## Conclusões

Nesta coorte retrospectiva, a localização lateral esquerda da VA e a maior idade estiveram independentemente associadas a maior prevalência de FA clínica. Em contraste, a localização posterior da VA e o sexo masculino estiveram associados a maior probabilidade de FA induzida no EEF.

## Data Availability

Todo o conjunto de dados que dá suporte aos resultados deste estudo está disponível mediante solicitação ao autor correspondente.
