# Supplementary material for: Associação entre a Ocorrência de Fibrilação Atrial e a Síndrome de Wolff-Parkinson-White: A Predição é Possível?
Source: Arq Bras Cardiol. 2026 Jun 10;123(5):e20260001. [Article in Portuguese] doi: 10.36660/abc.20260001 (PMC13399060; doi:10.36660/abc.20260001)
Supplement: Translation

# Translation: Association Between Atrial Fibrillation Occurrence and Wolff-Parkinson-White Syndrome: Is Prediction Possible?

**DOI:** 10.36660/abc.20260001i

**Authors:** Afonso Dalmazio Souza Mario, Eduardo Pelegrineti Targueta, Francisco Carlos da Costa Darrieux, Alexandra Regia Dantas Brigido, Alberto Pereira Ferraz, Muhieddine Omar Chokr, Cristiano F. Pisani, Mauricio Ibrahim Scanavacca

**Affiliations:** 1 São Paulo SP Brazil Instituto do Coração do Hospital das Clínicas da Faculdade de Medicina da Universidade de São Paulo, São Paulo, SP – Brazil

**Keywords:** Atrial Fibrillation, Wolff-Parkinson-White Syndrome, Electrophysiology, Cardiac Arrhythmias

## Abstract

**Background:**

Patients with Wolff-Parkinson-White (WPW) syndrome frequently develop atrial fibrillation (AF); however, the factors associated with the occurrence of AF remain incompletely defined.

**Objectives:**

To evaluate the prevalence and associated factors of clinical AF and electrophysiological study (EPS)-induced AF in a large consecutive cohort of patients with WPW undergoing accessory pathway (AP) ablation.

**Methods:**

This retrospective analysis included 845 consecutive patients with WPW who underwent AP ablation at a single tertiary referral center. A standardized EPS protocol was applied throughout the study period. Study outcomes included clinical AF (documented by medical history or monitoring before EPS), EPS-induced AF, and any AF (composite outcome). Multivariable logistic regression models were used to estimate adjusted odds ratio (ORa) with 95% CIs. Statistical significance was set at p < 0.05.

**Results:**

Mean age was 33.2 ± 15.9 years, and 486 of 845 patients (57.5%) were male. Multiple APs were identified in 31 patients (3.7%). The most common AP locations were left lateral (327/845, 38.7%) and posterior (323/845, 38.2%). Clinical AF was present in 109 patients (12.9%), EPS-induced AF in 77 (9.1%), and any AF in 168 (19.9%). In multivariable analyses, a left lateral AP was independently associated with clinical AF (ORa, 2.31; 95% CI, 1.53-3.49; p < 0.001), whereas a posterior AP was associated with EPS-induced AF (ORa, 1.78; 95% CI, 1.07-2.91; p = 0.025). Female sex was associated with lower odds of EPS-induced AF (ORa, 0.56; 95% CI, 0.34-0.93; p = 0.025). Increasing age was independently associated with clinical AF (per-year ORa, 1.018; p = 0.007) and any AF (per-year ORa, 1.014; p = 0.009).

**Conclusion:**

In this large cohort of patients with WPW undergoing AP ablation, left lateral AP location and older age were independently associated with a higher prevalence of clinical AF. In contrast, posterior AP location and male sex were associated with a greater likelihood of EPS-induced AF.

## Introduction

Atrial fibrillation (AF) is one of the arrhythmic manifestations observed in patients with Wolff-Parkinson-White (WPW) syndrome.^1^ However, AF in this population is clinically relevant because of its potential association with sudden cardiac death.^2^ Several mechanisms have been proposed to explain the occurrence of AF in patients with WPW, including degeneration of atrioventricular reentrant tachycardia (AVRT) into AF, electrophysiological properties of the accessory pathway (AP), AP-related effects on atrial architecture, and intrinsic atrial myocardial vulnerability.

Electrical impulses reaching the atria through an AP during AVRT may trigger AF episodes. In addition, AVRT may increase atrial vulnerability through enhanced sympathetic activation and atrial fiber stretching caused by the hemodynamic changes associated with tachyarrhythmia.^3^ The reduction in AF incidence after AP catheter ablation further supports the role of the AP in the pathophysiology of this arrhythmia.^4,5^ Some clinical characteristics, including older age, male sex, and posteroseptal AP location, have been associated with a higher likelihood of AF occurrence.^6^

Expanding the available evidence regarding the occurrence of AF in patients with WPW is important and may contribute to future meta-analyses. Therefore, this study aimed to evaluate factors associated with the occurrence of AF in patients with WPW through an association study based on a historical cohort.

## Methods

### Study design and population

This retrospective, analytical association study (historical cohort) was conducted in accordance with the principles of the Declaration of Helsinki and was approved by the local institutional review board.

All consecutive patients with WPW syndrome who underwent AP ablation between January 2016 and August 2021 at a tertiary cardiology referral center were included (Figure 1). Inclusion criteria required completion of the ablation procedure. Indications for ablation included symptomatic AVRT or asymptomatic patients presenting high-risk conduction properties. This cohort was independent of any previously published dataset.

**Figure 1:** Flowchart of the study population with WPW syndrome. AF: atrial fibrillation; AP: accessory pathway; EPS: electrophysiological study; WPW: Wolff-Parkinson-White.

### Electrophysiological study protocol

Programmed atrial stimulation was performed from the proximal coronary sinus (CS) using drive cycle lengths of 600, 500, and 400 ms, with up to two atrial extrastimuli delivered to the effective refractory period or a minimum coupling interval of 200 ms, followed by decremental pacing. A standardized induction protocol was consistently applied throughout the study period.

The protocol was repeated after intravenous administration of isoproterenol (10-20 mcg bolus). Induced AF included both AF intentionally triggered during programmed stimulation and inadvertent initiation of AF during the procedure. AF was defined as ≥ 10 seconds of irregular atrial activity without organized atrial tachycardia (AT) or AVRT morphology. AF occurring exclusively during catheter manipulation was classified as induced AF only when reproducible with programmed atrial stimulation.

### Outcomes

The study outcomes included clinical AF, electrophysiological study (EPS)-induced AF, and any AF (composite outcome including either clinical or induced AF).

### Statistical analysis

Continuous variables are presented as mean ± standard deviation, whereas categorical variables are expressed as absolute (n) and relative (%) frequencies.

The frequency of each presentation of AF (clinical AF, EPS-induced AF, or any AF) was evaluated according to qualitative variables. Associations between categorical variables were assessed using the chi-square test or Fisher's exact test, as appropriate. Age distributions according to the occurrence of AF were compared using the unpaired Student's *t* test.

Odds ratios (ORs) for each AF outcome were initially estimated using univariable logistic regression models. Subsequently, multivariable logistic regression models were constructed to identify independent predictors of each AF outcome. Sex, age, and procedural characteristics with p < 0.20 in unadjusted analyses were entered into the multivariable models. Variables with p < 0.20 in the univariable analysis were selected according to established recommendations for logistic regression modeling to avoid premature exclusion of potentially relevant predictors.^7^

Data normality was assessed using the Shapiro-Wilk test, and all continuous variables demonstrated normal distribution. Statistical analyses were performed using IBM SPSS Statistics for Windows, version 22.0 (IBM Corp., Armonk, N.Y., USA). Microsoft Excel 2013 (Microsoft Corp., Redmond, Washington, USA) was used for data tabulation. A two-sided p < 0.05 was considered statistically significant.

## Results

The study cohort comprised 845 patients with WPW syndrome who underwent AP catheter ablation. Baseline characteristics are summarized in Table 1. Mean age was 33.2 ± 15.9 years, and 139 patients (16.4%) were younger than 18 years. Overall, 486 patients were male (57.5%).

**Table 1:** Baseline patient characteristics and procedural findings

Variable		Value
Patients		845
Female sex		359 (42.5)
Age range, years		33.2 ± 15.9
	< 18 years	139 (16.4)
	18-39 years	431 (51.0)
	40-59 years	216 (25.6)
	≥ 60 years	59 (7.0)
Multiple APs		31 (3.7)
Posterior AP		323 (38.2)
Left lateral AP		327 (38.7)
EPS-induced AF		77 (9.1)
Clinical AF		109 (12.9)
Any AF		168 (19.9)

Values are presented as n (%) or mean ± standard deviation. AF: atrial fibrillation; AP: accessory pathway; EPS: electrophysiological study.

The most common AP locations were left lateral (37.4%), posteroseptal (30.9%), and right lateral (12.4%) (Figure 2). Multiple APs were identified in 31 patients (3.7%).

**Figure 2:** Distribution of AP locations identified during EPS in 845 ablation procedures. Some patients had multiple APs, resulting in a total of 876 APs identified. A: aortic valve; AP: accessory pathway; M: mitral valve; P: pulmonary valve; T: tricuspid valve.

The prevalence of clinical AF was 12.9% (n = 109). The prevalence of EPS-induced AF was 9.1%.

The frequency of AF increased progressively across age groups regardless of the AF definition used. The prevalence of EPS-induced AF increased from 5.0% among patients aged < 18 years to 15.3% among those aged ≥ 60 years. Similarly, the prevalence of clinical AF increased from 9.4% to 18.6%, whereas the prevalence of any AF increased from 13.7% to 23.7% across the same age strata (Table 2).

**Table 2:** EPS-induced, clinical, and any atrial fibrillation according to age group

Age group, years	EPS-induced AF, n (%)		Clinical AF, n (%)		Any AF, n (%)		Total
	No	Yes	No	Yes	No	Yes	
< 18	132 (95.0)	7 (5.0)	126 (90.6)	13 (9.4)	120 (86.3)	19 (13.7)	139
18-39	390 (90.5)	41 (9.5)	382 (88.6)	49 (11.4)	346 (80.3)	85 (19.7)	431
40-59	196 (90.7)	20 (9.3)	180 (83.3)	36 (16.7)	166 (76.9)	50 (23.1)	216
≥ 60	50 (84.7)	9 (15.3)	48 (81.4)	11 (18.6)	45 (76.3)	14 (23.7)	59
Total	768 (90.9)	77 (9.1)	736 (87.1)	109 (12.9)	677 (80.1)	168 (19.9)	845

The age-stratified analysis demonstrated a progressive increase in AF frequency with advancing age across all AF definitions. AF: atrial fibrillation; EPS: electrophysiological study.

Sex and posterior AP location were significantly associated with EPS-induced AF in unadjusted analyses (p < 0.05). After multivariable adjustment, both variables remained independently associated with induced AF (Table 3). Female sex was associated with a 44% lower likelihood of induced AF compared with male sex, whereas posterior AP location was associated with a 78% higher likelihood of induced AF, independent of the other evaluated characteristics.

**Table 3:** EPS-induced atrial fibrillation according to patient and procedural characteristics and results of univariable and multivariable analyses

Variable	EPS-induced AF, n (%)		OR_u_	95% CI	p-value	OR_a_	95% CI	p-value
	No	Yes						
Female sex, n (%)	335 (93.3)	24 (6.7)	0.590	0.350-0.970	0.035	0.560	0.340-0.930	0.025
Age, years (mean ± SD)	32.9 ± 16.0	36.5 ± 14.8	1.014	0.999-1.029	0.060†	1.014	0.999-1.029	0.064
Multiple APs, n (%)	27 (87.1)	4 (12.9)	1.500	0.510-4.420	0.517*	—	—	—
Posterior AP, n (%)	283 (87.6)	40 (12.4)	1.850	1.160-2.970	0.009	1.780	1.110-2.860	0.017
Left lateral AP, n (%)	299 (91.4)	28 (8.6)	0.900	0.550-1.460	0.659	—	—	—

Adjusted analyses were performed using multivariable logistic regression.

* Fisher's exact test.

† Student's t test. AF: atrial fibrillation; AP: accessory pathway; EPS: electrophysiological study; SD: standard deviation; OR_a_: adjusted odds ratio; OR_u_: unadjusted odds ratio.

As shown in Table 4, both age and left lateral AP location were significantly associated with clinical AF in unadjusted and adjusted analyses (p < 0.05). Each additional year of age was associated with a 1.8% increase in the likelihood of clinical AF. In addition, patients with a left lateral AP had a 2.31-fold higher likelihood of clinical AF compared with those without this AP location, independent of the other evaluated characteristics.

**Table 4:** Clinical atrial fibrillation according to patient and procedural characteristics and results of univariable and multivariable analyses

Variable	Clinical AF, n (%)		OR_u_	95% CI	p-value	OR_a_	95% CI	p-value
	No	Yes						
Female sex, n (%)	313 (87.2)	46 (12.8)	0.990	0.660-1.480	0.949	0.930	0.620-1.420	0.748
Age, years (mean ± SD)	32.6 ± 15.6	37.7 ± 16.8	1.020	1.008-1.033	0.001†	1.018	1.005-1.031	0.007
Multiple APs, n (%)	28 (90.3)	3 (9.7)	0.720	0.210-2.400	0.787*	—	—	—
Posterior AP, n (%)	284 (87.9)	39 (12.1)	0.890	0.580-1.350	0.574	—	—	—
Left lateral AP, n (%)	264 (80.7)	63 (19.3)	2.450	1.630-3.690	< 0.001	2.310	1.530-3.490	< 0.001

Adjusted analyses were performed using multivariable logistic regression.

* Fisher's exact test.

† Student's t test. AF: atrial fibrillation; AP: accessory pathway; SD: standard deviation; ORa: adjusted odds ratio; ORu: unadjusted odds ratio.

Similar findings were observed for any AF (Table 5). After adjustment, each additional year of age increased the likelihood of any AF by 1.4%, whereas patients with a left lateral AP had a 1.65-fold higher likelihood of any AF compared with those without this AP location, independent of the remaining covariates.

**Table 5:** Any atrial fibrillation according to patient and procedural characteristics and results of univariable and multivariable analyses

Variable	Any AF, n (%)		OR_u_	95% CI	p-value	OR_a_	95% CI	p-value
	No	Yes						
Female sex, n (%)	296 (82.5)	63 (17.5)	0.770	0.550-1.090	0.144	0.740	0.520-1.050	0.088
Age, years (mean ± SD)	32.5 ± 15.8	36.3 ± 15.8	1.015	1.004-1.026	0.005†	1.014	1.004-1.025	0.009
Multiple APs, n (%)	26 (83.9)	5 (16.1)	0.770	0.290-2.030	0.594	—	—	—
Posterior AP, n (%)	253 (78.3)	70 (21.7)	1.200	0.850-1.690	0.305	—	—	—
Left lateral AP, n (%)	244 (74.6)	83 (25.4)	1.730	1.230-2.440	0.001	1.650	1.170-2.330	0.004

Adjusted analyses were performed using multivariable logistic regression.

* Fisher's exact test.

† Student's t test. AF: atrial fibrillation; AP: accessory pathway; SD: standard deviation; ORa: adjusted odds ratio; ORu: unadjusted odds ratio.

The main findings are summarized in Central Illustration. Sensitivity analyses excluding patients with multiple APs showed largely consistent results. The only notable change was that female sex became borderline significant for EPS-induced AF after exclusion of patients with multiple APs (adjusted OR, 0.617, 95% CI, 0.370-1.028; p = 0.064).

## Discussion

To the best of our knowledge, this represents the third largest contemporary cohort of patients with WPW syndrome undergoing AP catheter ablation reported to date.^8–10^

The mean age of our cohort was 33 years, which is older than the 19 years reported by Pappone et al.^9^ in 2014. This difference may reflect disparities in access to specialized arrhythmia referral centers in Brazil.

Multiple APs were identified in 3.7% of patients, a lower proportion than previously reported in other contemporary cohorts.^8–10^ Nevertheless, AP distribution was consistent with prior studies,^9^ with left lateral APs being the most common, followed by posteroseptal APs.

Our findings regarding the prevalence of EPS-induced AF were also comparable to those reported in prior research (9.1% vs 11.1%).^9^ Likewise, the prevalence of clinical AF in our cohort (12.9%) exceeded the 2%-4% prevalence reported for the general population but was similar to that observed in other WPW ablation registries (10% and 12.8%).^8,10,11^

In our cohort, posterior AP location was associated with a higher likelihood of EPS-induced AF, whereas left lateral AP location was associated with clinical AF. Several mechanisms may explain the association between AP location and the occurrence of AF. Anatomical differences in AP insertion may contribute to atrial electrical instability. Posterior APs, which often have an oblique insertion pattern, may alter atrial myocardial architecture and increase susceptibility to atrial arrhythmogenesis.^3^

With respect to left-sided APs, previous studies have described an association between left lateral APs and atrial double potentials (DPs). DPs are typically identified during AVRT recordings in the CS. A Polish cohort study published in 2017 demonstrated an association between atrial DPs, clinical AF, induced AF, and older age.^12^ In that study, patients with atrial DPs also exhibited higher heart rates during AVRT and greater electrical alternans.

One possible mechanism underlying this association is simultaneous activation toward both the distal CS, close to the upper left pulmonary vein or mitral annulus, and the proximal (ostial) CS, leading to activation of the atrioventricular node. Generating dual atrial wavefronts may be a factor in the initiation of AF. In addition, the CS has close anatomical relationships with the inferoposterior right atrium and posterior left atrium, structures known to participate in the maintenance of AF. Tachycardia cycle length may also contribute to atrial vulnerability, as shorter cycle lengths may facilitate AF initiation by shortening atrial refractory periods.

Similar to AVRT, ventricular ectopic beats with retrograde conduction have also been associated with the occurrence of AF. Previous evidence suggests that multiple APs increase AF risk, likely because they enhance retrograde atrial activation. However, this association was not observed in our cohort, possibly because of the relatively low prevalence of multiple APs in our population.

Antegrade conduction properties have also been linked to the occurrence of AF.^1^ Previous studies demonstrated a higher prevalence of AF in patients with manifest APs compared with concealed APs. In addition, AP electrophysiological characteristics, particularly shorter refractory periods, have been associated with a higher likelihood of AF.

We interpret EPS-induced AF as a physiological stress test reflecting atrial vulnerability rather than a deterministic predictor of spontaneous AF at the individual level. The divergence observed in our study, with clinical AF associated predominantly with left lateral APs and EPS-induced AF associated with posterior APs, may reflect distinct trigger and substrate mechanisms, including dual-wavefront left atrial dispersion versus adrenergically facilitated and electrically labile triggers near the CS and inferoseptal regions.

### Limitations of the study

This study has several limitations. First, because this was a single-center retrospective study, selection bias cannot be excluded despite the large sample size. Detailed information regarding ablation indication categories (symptomatic vs asymptomatic high-risk patients) was not consistently available, although all included patients underwent ablation for one of these indications. Manifest versus concealed AP status was also not systematically recorded and therefore could not be incorporated into the analyses. In addition, AVRT cycle length was not consistently documented throughout the study period, precluding analyses involving tachycardia rate.

The association between left lateral AP location and clinical AF should also be interpreted cautiously, particularly because left lateral APs represented the most prevalent AP location in our cohort and because age is a strong and universal determinant of AF risk. Finally, long-term follow-up after ablation was not available, limiting our ability to determine whether atrial vulnerability in WPW represents a transient electrophysiological phenomenon or an independent long-term substrate for future development of AF.

## Conclusions

In this retrospective cohort, left lateral AP location and older age were independently associated with a higher prevalence of clinical AF. In contrast, posterior AP location and male sex were associated with a greater likelihood of EPS-induced AF.

## Data Availability

All datasets supporting the results of this study are available upon request from the corresponding author.
